# Study on the antagonistic effects of koumiss on *Toxoplasma gondii* infection in mice

**DOI:** 10.3389/fnut.2022.1014344

**Published:** 2022-09-28

**Authors:** Xinlei Yan, Yufei Sun, Guangzhi Zhang, Wenying Han, Jialu Gao, Xiuli Yu, Xindong Jin

**Affiliations:** ^1^Food Science and Engineering College of Inner Mongolia Agricultural University, Hohhot, China; ^2^Institute of Animal Sciences, Chinese Academy of Agricultural Sciences, Beijing, China

**Keywords:** koumiss, *Toxoplasma gondii*, cytokines, intestinal microbiota, β-amyloid deposition, cyst counting

## Abstract

*Toxoplasma gondii* is an important food-borne zoonotic parasite, and approximately one-third of people worldwide are positive for *T. gondii* antibodies. To date, there are no specific drugs or vaccines against *T. gondii*. Therefore, developing a new safe and effective method has become a new trend in treating toxoplasmosis. Koumiss is rich in probiotics and many components that can alleviate the clinical symptoms of many diseases *via* the functional characteristics of koumiss and its regulation of intestinal flora. To investigate the antagonistic effect of koumiss on *T. gondii* infection, the model of acute and chronic *T. gondii* infection was established in this study. The survival rate, SHIRPA score, serum cytokine levels, brain cyst counts, β-amyloid deposition and intestinal flora changes were measured after koumiss feeding. The results showed that the clinical symptoms of mice were improved at 6 dpi and that the SHIRPA score decreased after koumiss feeding (*P* < 0.05). At the same time, the levels of IL-4, IFN-γ and TNF-α decreased (*P* < 0.001, *P* < 0.001, *P* < 0.01). There was no significant difference of survival rate between koumiss treatment and the other groups. Surprisingly, the results of chronic infection models showed that koumiss could significantly reduce the number of brain cysts in mice (*P* < 0.05), improve β-amyloid deposition in the hippocampus (*P* < 0.01) and decrease the levels of IFN-γ and TNF-α (*P* < 0.01, *P* < 0.05). Moreover, koumiss could influence the gut microbiota function in resisting *T. gondii* infection. In conclusion, koumiss had a significant effect on chronic *T. gondii* infection in mice and could improve the relevant indicators of acute *T. gondii* infection in mice. The research provides new evidence for the development of safe and effective anti-*T. gondii* methods, as well as a theoretical basis and data support for the use of probiotics against *T. gondii* infection and broadened thoughts for the development and utilization of koumiss.

## Introduction

*Toxoplasma gondii* is a worldwide intracellular protozoan parasite that can infect almost all warm-blooded animals and cause zoonotic diseases (1). *T. gondii* has a complex life cycle, including tachyzoites, bradyzoites, oocysts, schizonts and gametocytes. Humans are mainly infected by ingesting food (cysts) and drinking water (oocysts) contaminated by *T. gondii* or undercooked or raw meat (cysts) containing tissue cysts (2). It causes toxoplasmosis after the host is infected with *T. gondii*. Acute toxoplasmosis develops when tachyzoites invade and replicate within the cells. It can result in fatal encephalitis, pneumonitis and myocarditis. The severity of infection is associated with the genotype of the parasite strain, the form of infection and host immunity (3). Tachyzoites spread to brain and skeletal muscle through the blood, convert into bradyzoites under the function of autoimmunity, and subsequently persist for a long time as dormant cysts (4, 5). The conversion from tachyzoites to bradyzoites is a sign of infection in the chronic phase (4).

It is estimated that more than 80% of immunocompetent individuals are asymptomatic after infection with *T. gondii* (6). Therefore, chronic toxoplasmosis was previously considered benign (7). As a major neurotropic pathogen, *T. gondii* has a higher affinity for the central nervous system than other organs (8). Studies have shown that *T. gondii* is closely linked with epilepsy (9), Alzheimer’s disease (10) and schizophrenia (11). Furthermore, *T. gondii* infection could be responsible for cognitive dysfunction and increased suicidal behaviors (12, 13). Another study suggested that the relative abundance of harmful bacteria increased in the intestine in mice infected with *T. gondii* (14). Notably, dormant cysts can be reactivated and lead to severe consequences once host immunity is defective (15). However, there are no specific vaccines or drugs against *T. gondii* thus far. Sulfonamides are beneficial to treat acute toxoplasmosis but ineffective for chronic toxoplasmosis accompanied by side effects. Consequently, a new, secure and effective method should be sought to alleviate or ameliorate *T. gondii* infection.

Koumiss is a kind of traditional fermented milk product made from mare’s milk, which is widely popular in Russia, Mongolia, eastern Europe and central Asia. The koumiss culture can be traced back to the Han dynasty in China. Koumiss has a large number of nutrients, including all the essential amino acids needed by human beings, such as proline, lysine, tyrosine, valine and leucine (16). Moreover, koumiss has more essential fatty acids than milk, which is good for human health (17). The ratio of casein to whey is 1:1 in koumiss, which is close to that in human milk (18). In addition to its high nutritional value, koumiss also has excellent therapeutic potential due to its anticarcinogenic and antioxidative properties, antibacterial properties and intestinal enlargement (16). Koumiss was reported as a therapeutic beverage against tuberculosis in 1861 (19). Those of Mongolian ethnicity have developed “koumiss therapy,” which combines traditional Mongolian medicine with koumiss used in the clinical treatment of intestinal indigestion, hypertension, tuberculosis, and other cases (20, 21). In addition, due to its rich probiotics and good safety, koumiss has been used instead of antibiotics to inhibit the growth of harmful bacteria and maintain the natural flora balance (22, 23). Modern medical research confirmed that koumiss could alleviate chronic atrophic gastritis and hyperlipidemia by modulating gut microbiota (20, 24).

To explore the effects of koumiss on *T. gondii* infection, BALB/c mice were inoculated with *T. gondii* cysts and gavaged orally with koumiss. The survival rate, serum cytokine levels, SHIRPA score, number of brain cysts and 16S rRNA gene sequence of intestinal microbiota were determined. This study reveals the effects of koumiss consumption in treating toxoplasmosis by regulating gut microbiota for the first time and provides a theoretical basis and data support for developing a novel treatment against *T. gondii* infection.

## Materials and methods

### Animals and parasites

Female BALB/c mice, 6 weeks old, weighing 15–18 g, were purchased from SPF (Beijing) Biotechnology Co., Ltd. The mice were given food and water arbitrarily and housed under a 12 h light/dark cycle. The animal protocols were reviewed and approved by the Inner Mongolia Agricultural University Laboratory Animal Welfare and Animal Experimental Ethical Inspection Committee (NND2021069). All experiments were performed according to the relevant guidelines and regulations. The *T. gondii* PRU strain was obtained from the National Animal Protozoa Laboratory of China Agricultural University, passaged and preserved serially in Kunming mice (25). Koumiss samples and fresh mare’s milk samples obtained from the Abaga banner of Xilin Gol League were used in their original form (fermentation temperature: 18–20°C, fermentation time: 72 h, pH value: 3.88).

### Acute infection

Forty-eight BALB/c mice were randomly divided into the acute control group (AC, *n* = 12), acute infection group (AI, *n* = 12), acute koumiss group (AK, *n* = 12) and acute mare’s milk group (AM, *n* = 12). The AI, AK and AM groups were orally inoculated with 200 μL PBS containing 50 cysts, while the AC group was inoculated with the same volume of PBS alone. The AC, AI, AK and AM groups were gavaged with 100 μL PBS, PBS, koumiss and mare’s milk respectively everyday. The mice were used to calculate the survival rate after 6 dpi.

### Chronic infection

One hundred and forty-four BALB/c mice were randomly separated into the chronic control group (CC, *n* = 36), chronic infection group (CI, *n* = 36), chronic koumiss group (CK, *n* = 36) and chronic mare’s milk group (CM, *n* = 36). The CI, CK and CM groups were infected with 3 cysts in 200 μL PBS, and the C group was infected with the same volume of PBS alone. The CC, CI, CK, and CM groups were gavaged with 100 μL PBS, PBS, koumiss and mare’s milk respectively every 2 days.

### Serum cytokine level measurement

The blood of mice was collected by the eyeball removal method at 6 d post-infection (dpi) in the acute infection, and chronic infection group collected at 14 dpi. Serum was separated at 1,000 × g for 20 min and stored at −20°C. Enzyme-linked immunosorbent assay (ELISA) kits (Tiangen, Beijing. Production lot number: 03/2021) were used to determine the serum levels of IL-4, IL-10, IFN-γ, and TNF-α. All operations were carried out in accordance with the manufacturer’s instructions.

### Clinical score

The modified SHIRPA index was used to assess the clinical symptoms of mice infected with *T. gondii* (26). Acute infection mice were assayed at 6 dpi. The modified SHIRPA index provides quantitative data for individual performance, which includes a series of individual tests. The tests were performed in the following order, and one point was given for conformity: piloerection, abdominal writhing, weight loss, diarrhea, lacrimation, palpebral closure, moving speed, reflexive escape from touch, spontaneous tremors, reduced grip strength, hunched posture, and changes in respiration rate (hyperventilation) (27). The control group was regarded as having no changes in the clinical score.

### *T. gondii* cyst counting

Mice were euthanized by cervical dislocation, and the brain tissue was separated and homogenized with 3 mL of PBS. A 20 μL sample of each homogenate was placed into the blood count chamber, and the intact cysts were counted under a light microscope for three times. The average value was calculated for the final result.

### β-amyloid deposition assay

On 42 dpi, the chronic infection mice were euthanized, and the hippocampus were separated and used in the following trial. Immunohistochemical staining was used to determine β-amyloid deposition in the hippocampus. The hippocampal tissue was fixed with 4% paraformaldehyde, embedded in paraffin wax and sliced (4 μM). Rabbit anti-β-amyloid 1-40 (CT) antibody (Bioss, USA) was diluted 200 times to treat the slices, and then the samples were DAB-developed, dehydrated, and sealed transparently. A Leica microscope was used to observe and photograph the sections.

### 16S rRNA gene sequence measurement

Three mice were selected randomly from each group at 6 dpi in the acute infection, and six mice were selected at random from each group at 7, 14, 21, 28, 35, and 42 dpi in the chronic infection. Feces were collected aseptically within 2 h, treated with liquid nitrogen and stored at −80° until the next experiment. Genomic DNA was extracted from the feces using the cetyltrimethylammonium ammonium bromide (CATB) method. Agarose gel electrophoresis was used to determine the purity and concentration of genomic DNA. The 16S rRNA genes were amplified with specific primers with barcodes. The PCR primers and conditions were performed as described previously (28). The PCR products were determined by 2% agarose gel electrophoresis and purified with a Qiagen Gel Extraction Kit (Qiagen, Germany).

Libraries were constructed using the TruSeq^®^ DNA PCR-Free Sample Preparation Kit (Illumina, USA) and sequenced with high-throughput sequencing technology on the Illumina NovaSeq sequencing platform. Each sample was measured three times. All effective tags of samples were clustered with 97% similarity into operational taxonomic units (OTUs). The taxonomic information was obtained by species annotation analysis, and the community composition of the samples was counted individually at each classification level, including the Kingdom, Phylum, Class, Order, Family, Genus and Species levels.

### Statistical analysis

GraphPad Prism software was used for data analyses and plots. Kyoto Encyclopedia for Genes and Genomics (KEGG) classifications were performed with the Tax4Fun software package. All data are expressed as mean ± standard deviation (SD). *P* < 0.05 was considered a significant difference.

## Results

### The survival rate of mice with acute *T. gondii* infection

**FIGURE 1:**
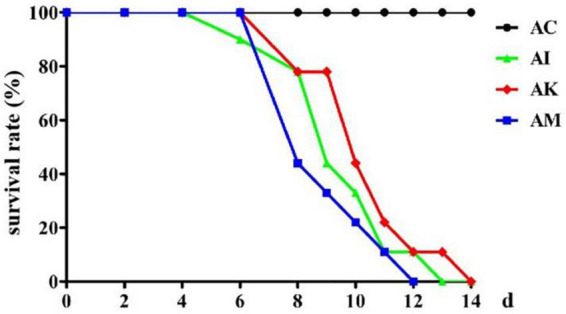
The survival rate of mice with acute *Toxoplasma gondii* infection. AC: no *T. gondii* + PBS; AI: 50 *T. gondii* cysts + PBS; AK: 50 *T. gondii* cysts + koumiss; AM: 50 *T. gondii* cysts + mare’s milk. All data are expressed as mean ± SD.

Mice in the AI, AK, and AM groups were inoculated with 50 cysts, and mice in the AC group received no treatment. The AC, AI, AK, and AM groups were gavaged daily with PBS, PBS, koumiss and mare’s milk, respectively. The survival rate was measured, and the results showed that AC resulted in no deaths, and the other groups all died (Figure 1). Koumiss did not increase the survival rate of mice with acute *T. gondii* infection.

### Effect of koumiss on the SHIRPA scores of mice with acute *T. gondii* infection

**FIGURE 2:**
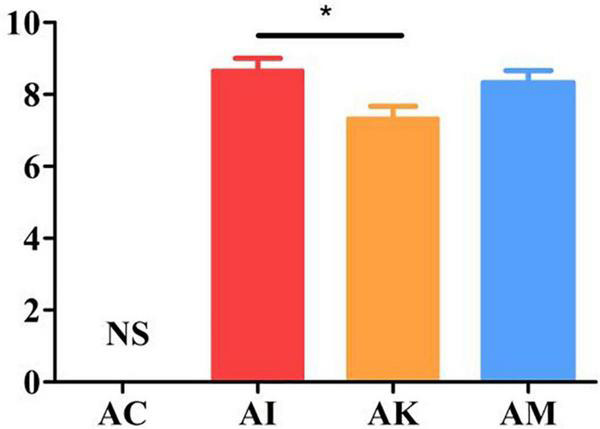
The SHIRPA scores of mice with acute *T. gondii* infection at 6 dpi. The control group (AC) was regarded as having no changes in the clinical score. AC: no *T. gondii* + PBS; AI: 50 *T. gondii* cysts + PBS; AK: 50 *T. gondii* cysts + koumiss; AM: 50 *T. gondii* cysts + mare’s milk. All data are expressed as mean ± SD. *P < 0.05 and N.S., no significance.

Mice showed a series of clinical symptoms after infection with *T. gondii*, including piloerection, palpebral closure, spontaneous tremors and hunched posture. At 6 dpi, the modified SHIRPA index was used to assess the clinical symptoms of the mice. The AC group was regarded as having no changes in the clinical score. The AI, AK and AM groups were evaluated on the basis of their behaviors. The results suggested that there was a significant difference between the AI and AK groups (*P* < 0.05). However, there was no difference between the AI and AM groups (Figure 2). This result indicated that koumiss treatment could improve the clinical symptoms of mice at 6 dpi with acute *T. gondii.* infection.

### Effect of koumiss on the serum cytokine levels of mice with acute *T. gondii* infection

**FIGURE 3:**
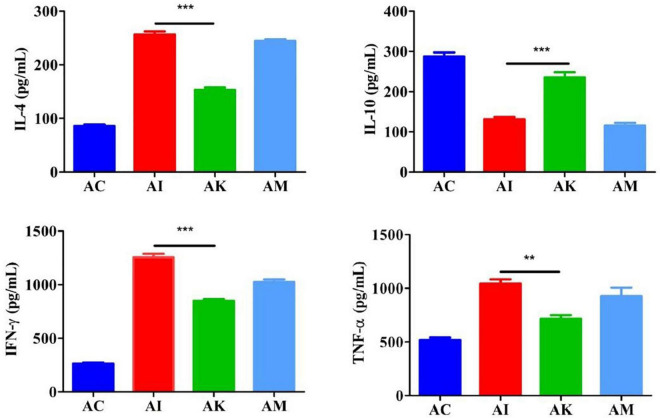
Serum levels of IL-4, IL-10, IFN-γ, and TNF-α in mice with acute *T. gondii* infection at 6 dpi. AC: no *T. gondii* + PBS; AI: 50 *T. gondii* cysts + PBS; AK: 50 *T. gondii* cysts + koumiss; AM: 50 *T. gondii* cysts + mare’s milk. All data are expressed as mean ± SD. **P < 0.01; ***P < 0.001.

ELISA was used to determine the serum cytokine levels according to the manufacturer’s instructions. The results showed that the levels of IL-4, IFN-γ and TNF-α increased, and the levels of IL-10 decreased after infection with *T. gondii* (Figure 3). There were significant differences between the AI and AK groups in the levels of IL-4, IFN-γ and TNF-α (*P* < 0.001, *P* < 0.001, *P* < 0.001). There was a higher level of IL-10 in the AK group than in the AI group (*P* < 0.01).

### Effect of koumiss on the gut microbiota of mice with acute *T. gondii* infection

**FIGURE 4:**
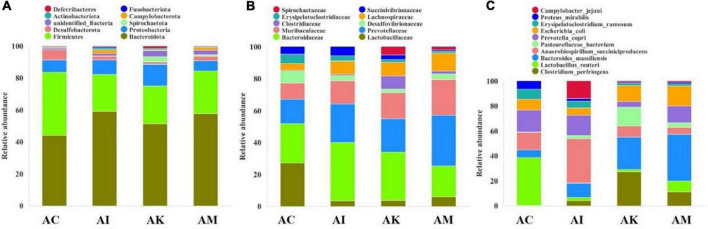
The intestinal microbiota composition changes of mice with acute *T. gondii* infection at 6 dpi. AC: no *T. gondii* + PBS; AI: 50 *T. gondii* cysts + PBS; AK: 50 *T. gondii* cysts + koumiss; AM: 50 *T. gondii* cysts + mare’s milk; **(A–C)** indicate phylum, family and species levels, respectively. All data are expressed as mean ± SD.

Feces were collected aseptically within 2 h on 6 dpi. The 16S rRNA gene sequences of gut flora were determined by high-throughput sequencing technology. The intestinal flora composition changes were analyzed at the phylum, family and species levels (Figure 4). The focus of analysis was the community structure of the gut microbiota whose abundances were ranked in the top 10. The species composition of the AC group consisted of *Bacteroidota* (43.8%), *Firmicutes* (39.2%), *Proteobacteria* (7.9%), *Desulfobacterota* (6.2%), *Campylobacterota* (0.6%), *Actinobacteriota* (0.6%) and others at the phylum level (Figure 4A). After infection with *T. gondii*, the relative abundance of *Bacteroidota* decreased, and *Firmicutes* increased in the AI, AK, and AM groups. At the family level, the relative abundances of *Lactobacillaceae* and *Desulfovibrionaceae* decreased in mice infected with *T. gondii* (Figure 4B). Moreover, there was an increasing trend in *Prevotellaceae*, *Muribaculaceae*, *Clostridiaceae*, and *Lachnospiraceae* in the AI, AK, and AM groups. There was a downwards trend in *Lactobacillus reuteri* in mice infected with *T. gondii* at the species level (Figure 4C).

### Effect of koumiss on the serum cytokine levels of mice with chronic *T. gondii* infection

**FIGURE 5:**
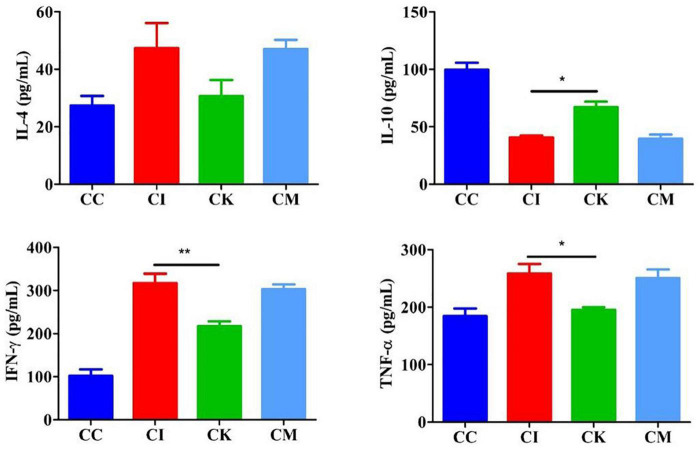
Serum levels of IL-4, IL-10, IFN-γ, and TNF-α in mice with chronic *T. gondii* infection at 14 dpi. CC: no *T. gondii* + PBS; CI: 3 *T. gondii* cysts + PBS; CK: 3 *T. gondii* cysts + koumiss; CM: 3 *T. gondii* cysts + mare’s milk. All data are expressed as mean ± SD. *P < 0.05; **P < 0.01.

Mice in the CI, CK, and CM groups were inoculated with 3 cysts and treated with PBS, koumiss and mare’s milk. The CC group was inoculated and treated with PBS alone. On 14 dpi, blood was obtained from eyeballs and centrifuged at 1,000 × *g* for 20 min. ELISA was used to determine the serum levels of IL-4, IL-10, IFN-γ, and TNF-α. The results showed that the levels of IL-4, IFN-γ, and TNF-α increased, and IL-10 decreased after infection with *T. gondii* (Figure 5). There was no difference in the levels of IL-4 among the different groups. Compared with the CI group, there were significant decreases in the levels of IFN-γ and TNF-α in the CK group (*P* < 0.01, *P* < 0.05). Moreover, there was a significant difference in the levels of IL-10 between the CI and CK groups (*P* < 0.05).

### Effect of koumiss on brain cyst counts in mice with chronic *T. gondii* infection

**FIGURE 6:**
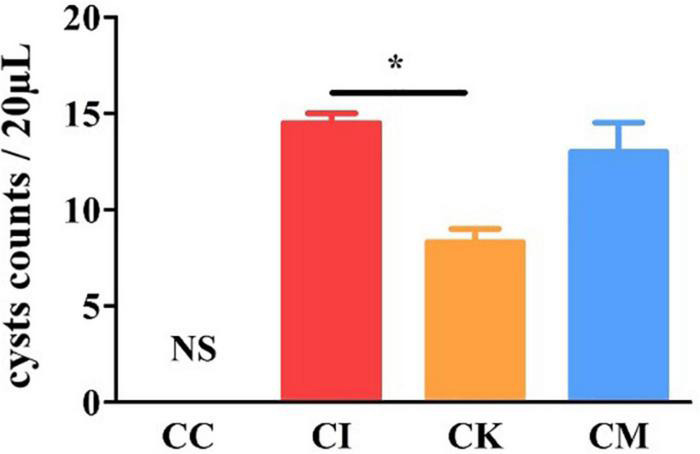
The brain cyst count in mice with chronic *T. gondii* infection at 42 dpi. CC: no *T. gondii* + PBS; CI: 3 *T. gondii* cysts + PBS; CK: 3 *T. gondii* cysts + koumiss; CM: 3 *T. gondii* cysts + mare’s milk. All data are expressed as mean ± SD. *P < 0.05 and N.S., no significance.

Brain cyst counting is the most direct and important index to reflect the chronic infection of *T. gondii*. The mice were euthanized by cervical dislocation, and brain tissue was homogenized with PBS at 42 dpi. Each sample was counted three times under a light microscope. The results showed that there was a significant difference in cyst counts between the CI and CK groups (*P* < 0.05) (Figure 6). There was no difference in cyst counts between the CI and CM groups. This indicated that koumiss could reduce the brain cyst count of mice chronically infected with *T. gondii*.

### Effect of koumiss on β-amyloid deposition in mice with chronic *T. gondii* infection

**FIGURE 7:**
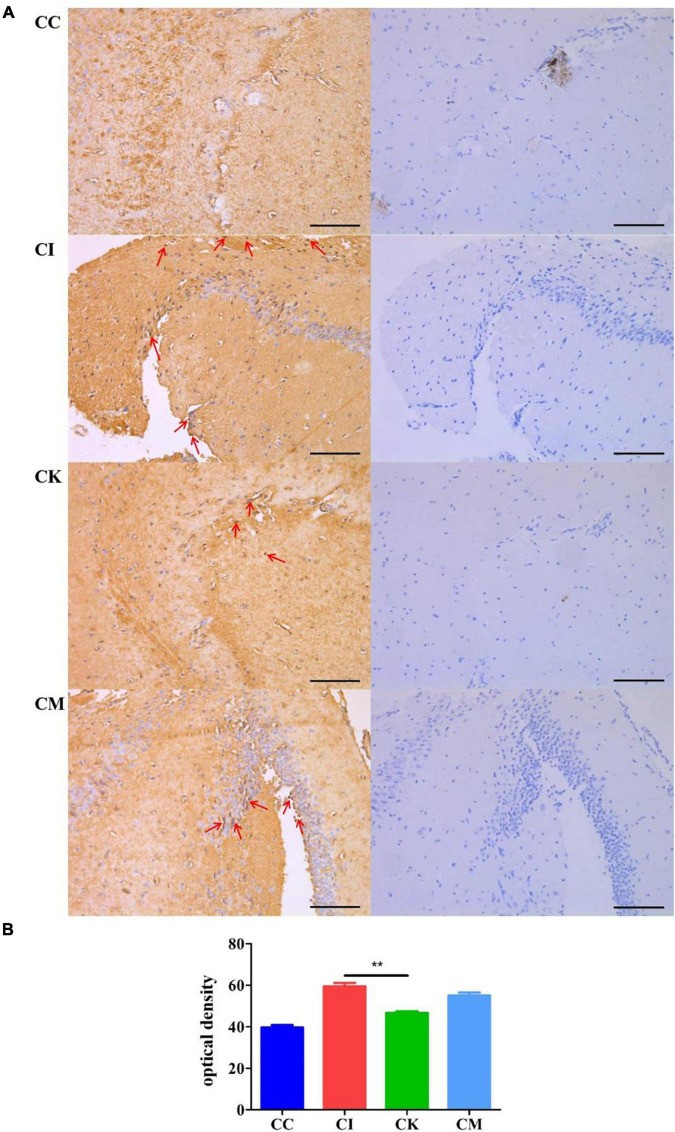
β-amyloid deposition in mice with chronic *T. gondii* infection at 42 dpi. CC: no *T. gondii* + PBS; CI: 3 *T. gondii* cysts + PBS; CK: 3 *T. gondii* cysts + koumiss; CM: 3 *T. gondii* cysts + mare’s milk; **(A)** The immunohistochemical staining results of the CC, CI, CK and CM groups. Red arrows represent brownish yellow particles caused by amyloid deposition. **(B)** The optical density value of different treatment groups. **P < 0.01. All data are expressed as mean ± SD. Scale bars: 100 μm.

When immunocompetent individuals are infected with *T. gondii*, it can form a recessive infection in the body. This recessive infection is closely related to anomalous cognitive behaviors, including schizophrenia and Alzheimer’s disease. β-amyloid plaques caused by amyloid deposition in the hippocampus are indicative of pathological changes in Alzheimer’s disease. In this study, immunohistochemical staining was used to determine the β-amyloid deposition in the hippocampus of mice chronically infected with *T. gondii* at 42 dpi. After immunohistochemical staining was performed, there were brownish yellow particles in the cytoplasm of neurons in the positive samples, while there were no brownish yellow particles in the negative samples. The results suggested that the CC group had no brownish yellow particles and that the CI, CK and CM groups showed many brownish yellow particles (Figure 7A), indicating that *T. gondii* infection resulted in β-amyloid deposition in the hippocampus of mice. Compared with the CI group, the CK group had a lower optical density (OD) value (*P* < 0.01) (Figure 7B). The optical density value is connected to the amount of dyeing. The greater the amount of dyeing, the higher the optical density value. Therefore, the results proved that there were fewer amyloid plaques in the CK group, which indicates that koumiss could reduce β-amyloid deposition.

### Effect of koumiss on the gut microbiota in mice with chronic *T. gondii* infection

**FIGURE 8:**
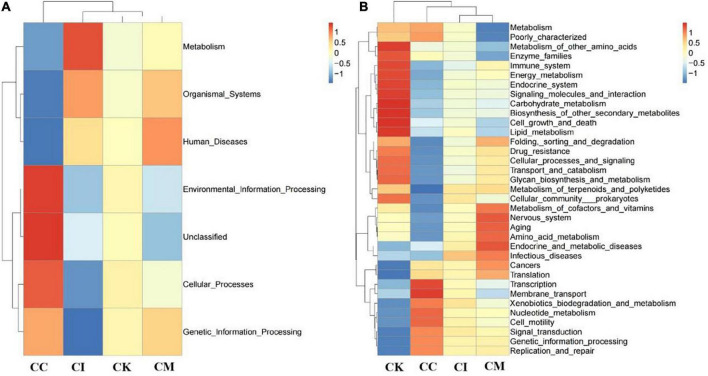
Effect of koumiss on the microbiota functional profile in mice chronically infected with *T. gondii*. **(A)** Level 1. **(B)** Level 2. CC: no *T. gondii* + PBS; CI: 3 *T. gondii* cysts + PBS; CK: 3 *T. gondii* cysts + koumiss; CM: 3 *T. gondii* cysts + mare’s milk. All data are expressed as mean ± SD.

Feces were collected aseptically, and the 16S rRNA gene sequences were determined with high-throughput sequencing technology. Tax4Fun analysis is often performed to predict the function of intestinal microbiota and environmental samples. Its core content is comparing 16S rRNA gene sequencing data with the KEGG database to achieve functional annotation. The results of this study showed that the CK group had more bacteria functioning in metabolism than the CC, CI and CM groups at 14 dpi, such as carbohydrate metabolism, energy metabolism and lipid metabolism (Figure 8A). Moreover, there was also an obvious increase in drug resistance, immune system and enzyme families in the CK group at 14 dpi. Membrane transport in the CK group showed a downwards trend compared with that in the CC group at 14 dpi (Figure 8B).

## Discussion

*Toxoplasma gondii* is one of the most successful pathogens in nature, it can infect a wide range of mammals and birds and cause potentially fatal diseases in humans (29). To date, there are no specific drugs and vaccines against *T. gondii*. Koumiss is rich in probiotics and various functional components that has been used to alleviate the clinical symptoms of many diseases due to its functional characteristics and regulation of intestinal flora. In this study, the effect of koumiss on *T. gondii* infection in BALB/c mice was explored.

Mice were inoculated with 50 PRU strain cysts to establish a model of acute *T. gondii* infection and were gavaged with koumiss at a dose of l00 μL/d. The survival rate, SHIRPA score, serum cytokine levels and changes in gut microbiota were measured. The results showed that all mice infected with *T. gondii* were dead at 14 dpi. No significant difference in survival rate with koumiss treatment compared to the AI group. However, after koumiss feeding, the levels of IL-4, IFN-γ, and TNF-α in the AK group were lower than those in the AI group. When the hosts were infected with *T. gondii*, IFN-γ and TNF-α were released to resist the pathogens. Excessive release of IFN-γ and TNF-α and disruption of the Th1/Th2 balance will result in pathological immunoreactions. This indicated that koumiss could play an important role in inhibiting the excessive secretion of proinflammatory factors.

In addition, the SHIRPA score was used to evaluate the behaviors of mice acutely infected with *T. gondii*. The results showed that there was a significant reduction in the AK group compared with the AI group, suggesting that koumiss can improve the clinical performance of mice acutely infected with *T. gondii*. Moreover, the results proved that koumiss influenced the gut microbiota composition of mice infected with *T. gondii*. The most important finding is that the relative abundance of *Lactobacillus reuteri* decreased in mice with acute *T. gondii* infection. *L. reuteri* naturally exists in the intestines of almost all vertebrates and mammals, has strong adhesion to the intestinal mucosa, and can improve the intestinal flora and prevent the colonization of harmful bacteria. The antibacterial substance produced by *L. reuteri* has an excellent inhibitory effect on the growth of bacteria, fungi and parasites (30). Previous studies have shown that *L. reuteri* combined with other probiotics can inhibit 80% of *Eimeria tenella* invasion of bovine kidney cells *in vitro* (31). Relevant studies have found that the probiotic *L. reuteri* KUB-AC5 can rapidly colonize the intestinal tract of mice and reduce the intestinal inflammation and systemic transmission of mice caused by Salmonella infection (32). In addition, *L. reuteri* was proven to inhibit the colonization of *Cryptosporidium parvum* in the intestinal epithelium of immunodeficient mice (33). Therefore, *L. reuteri* was considered to potentially inhibit the colonization of *T. gondii* in the host. Concerning the effect of koumiss on mice with acute *T. gondii* infection, the study results showed that koumiss did not increase the survival rate of mice but improved the relevant indicators, including the serum cytokine levels and SHIRPA score. To guarantee the establishment of an acute *T. gondii* infection model in mice, mice were gavaged with koumiss for a relatively short period of time (6 d), which may account for the experimental results.

To further explore the effect of koumiss on *T. gondii* infection, a chronic *T. gondii* infection model was established, and mice were gavaged with koumiss every 2 days. Then, the serum cytokine levels, brain cyst counts, β-amyloid deposition and intestinal flora changes were determined. The results showed that the levels of IL-4, IFN-γ and TNF-α increased, while the levels of IL-10 decreased at 14 dpi. IFN-γ and TNF-α are important cytokines against *T. gondii* infection that play an important role in the initial stage of acute infection and chronic infection (34). Koumiss can improve intestinal immune function by increasing the number of leukocytes, repairing the tissue structure of the spleen and thymus, and increasing the CD4^+^/CD8^+^ ratio in immunosuppressed rats (35). Therefore, after infection with *T. gondii*, the levels of IFN-γ and TNF-α increased to resist *T. gondii* infection. The decrease in the levels of IL-10 maintained the Th1/Th2 balance. After feeding on koumiss, the levels of IFN-γ and TNF-α decreased, and the levels of IL-10 increased, proving that koumiss could reduce the levels of proinflammatory factors and enhance the levels of anti-inflammatory factors to maintain the Th1/Th2 balance.

The brain cysts were counted at 42 dpi to determine the presence of tissue cysts in the brain (36). The results showed that there was a significant reduction in the CK group compared with the CI group, demonstrating that koumiss could reduce the brain cyst counts of mice with chronic *T. gondii* infection. A study showed that chronic *T. gondii* infection can change or subvert the activity of neurons and permanently affect the behavior of the host (37). Therefore, β-amyloid deposition in the hippocampus was measured with immunohistochemical staining. β-amyloid plaques produced by amyloid deposition in the brain are characteristic pathological changes of Alzheimer’s disease (38). Previous study has shown that fermented milk of *Lactobacillus helveticus* IDCC3801 reduced β-amyloid and improved memory deficit (39). The results showed that chronic *T. gondii* infection caused β-amyloid deposition in the hippocampus of mice, while lactic acid bacteria enriched in koumiss treatment may improved β-amyloid deposition. However, the cognitive behavior changes caused by *T. gondii* infection and the effects of koumiss require further testing.

Furthermore, gut microbiota changes were determined with high-throughput sequencing technology. The differences of microbiota structure were caused by strains of different genotypes (40). The results showed that koumiss could increase the relative abundance of bacteria functioning in metabolism at 14 dpi, such as carbohydrate metabolism and lipid metabolism. The glycolysis pathway plays an important role in the *T. gondii* tachyzoite stage because it provides a carbon source for fatty acid synthesis and drives *T. gondii* invasion of host cells (41). A study suggested that koumiss had prominent functions in carbohydrate metabolism and amino acid metabolism (42). Therefore, koumiss may inhibit the growth of *T. gondii* by regulating carbohydrate metabolism. In addition, lipids also participate in innate immunity and may play an important role in controlling *T. gondii* infection (43). Koumiss consumption caused significant changes in metabolites involving in numerous metabolic pathway, including biosynthesis of primary and secondary bile acid, as well as metabolism of unsaturated fatty acid, linoleic acid and arachidonic acid (44). The study also demonstrated that koumiss was related to fat synthesis and metabolism (18), which might be another important impact factor against *T. gondii* infection.

In this study, we explored the effect of koumiss on mice with acute and chronic *T. gondii* infection. Although feeding koumiss did not improve the survival rate of acute infection in mice, related indicators were improved, such as the SHIRPA score, cytokine level and intestinal flora. Moreover, koumiss had an obvious antagonistic effect on chronic *T. gondii* infection in mice, which was reflected in the reduction in brain cyst counts, the reduction in brain tissue inflammatory reactions and the increase in the relative abundance of certain bacteria related to the inhibition of *T. gondii* infection. This study provided a new theoretical basis and data support for the remission and treatment of *T. gondii* infection as well as novel ideas for research on the role of probiotics in resisting intracellular pathogen infection.

## Data availability statement

The data presented in this study are deposited in the NCBI repository (https://www.ncbi.nlm.nih.gov/), accession number: PRJNA868501.

## Ethics statement

The animal study was reviewed and approved by the Inner Mongolia Agricultural University Laboratory Animal Welfare and Animal Experimental Ethical Inspection Committee (NND2021069).

## Author contributions

XYan and YS: study concept and design, analysis, and interpretation of data. YS, XJ, WH, JG, and XYu: samples collection and experiment. YS and GZ: manuscript writing. XYan: manuscript review and editing, supervision, funding acquisition, and project administration. All authors contributed to the article and approved the submitted version.
